# Fitness Consequences of Northward Dispersal as Possible Adaptation to Climate Change, Using Experimental Translocation of a Migratory Passerine

**DOI:** 10.1371/journal.pone.0083176

**Published:** 2013-12-11

**Authors:** Claudia Burger, Andreas Nord, Jan-Åke Nilsson, Emmanuelle Gilot-Fromont, Christiaan Both

**Affiliations:** 1 Animal Ecology Group, Centre for Ecological and Evolutionary Studies, University of Groningen, Groningen, The Netherlands; 2 Department of Biology, Section for Evolutionary Ecology, Lund University, Lund, Sweden; 3 VetAgro Sup, Campus Vétérinaire de Lyon, Santé Publique Vétérinaire, Université de Lyon, Marcy l’Etoile, France; 4 Université de Lyon, Université Lyon 1 UMR5558 Laboratoire de Biométrie et Biologie Evolutive, Villeurbanne, France; University of Milan, Italy

## Abstract

Climate change leads to rapid, differential changes in phenology across trophic levels, often resulting in temporal mismatches between predators and their prey. If a species cannot easily adjust its timing, it can adapt by choosing a new breeding location with a later phenology of its prey. In this study, we experimentally investigated whether long-distance dispersal to northern breeding grounds with a later phenology could be a feasible process to restore the match between timing of breeding and peak food abundance and thus improve reproductive success. Here, we report the successful translocation of pied flycatchers (*Ficedula hypoleuca*) to natural breeding sites 560 km to the Northeast. We expected translocated birds to have a fitness advantage with respect to environmental phenology, but to potentially pay costs through the lack of other locally adapted traits. Translocated individuals started egg laying 11 days earlier than northern control birds, which were translocated only within the northern site. The number of fledglings produced was somewhat lower in translocated birds, compared to northern controls, and fledglings were in lower body condition. Translocated individuals were performing not significantly different to control birds that remained at the original southern site. The lack of advantage of the translocated individuals most likely resulted from the exceptionally cold spring in which the experiment was carried out. Our results, however, suggest that pied flycatchers can successfully introduce their early breeding phenotype after dispersing to more northern areas, and thus that adaptation through dispersal is a viable option for populations that get locally maladapted through climate change.

## Introduction

As a consequence of climate warming, the timing of many seasonal events has advanced, but trophic levels often differ in the speed and extent of advancement [1]. This can result in temporal mismatches between predators and their prey [2,3]. Important life-cycle events which determine individual fitness can be affected by such mismatches: It is, for example, assumed that timing of breeding in many bird species should coincide with maximum food abundance [4]. Mismatches with the food peak were therefore held responsible for reduced fitness and population declines in some bird species [2,5]. 

Rapid environmental changes can make it difficult for some species to adapt locally because the speed of micro-evolution is too low or because of a lack of genetic variation in the population [6]. Instead, individuals experiencing such rapid change may respond by dispersing northwards, to environments where conditions match their adaptations better than at the natal location [7]. By dispersing over a long distance to the North, individuals could escape negative fitness consequences of local mistiming [8]. Evidence for conditional dispersal, e.g. depending on reproductive success, exists, although this is described mostly for shorter dispersal distances [9]. Long-distance dispersal could have similar causes, but could also be solely the result of a stochastic process, related to the distribution of natal dispersal distances in long-distance migrants [10]. 

In addition to the direct fitness consequences of long-distance dispersal, an earlier breeding phenotype or eventually new genes might be introduced into a northern population on which selection can act [11]. For a number of songbirds, heritabilities for laying dates within populations have been found to be rather low [2,12], but between populations, distant populations may differ genetically in timing of laying [13,14]. In a study on pied flycatchers (*Ficedula hypoleuca*), neutral genetic markers suggest only moderate genetic differentiation between the Dutch and Scandinavian populations, although at least the genetically determined dorsal coloration in males differs among these populations [15]. Apart from the possible genetic consequences of dispersal, individuals could show an adaptive phenotypic response in the sense that relatively early-born young also return and breed relatively early in the next year. Such carry-over effects would therefore be a likely mechanism to affect timing of reproduction [16]. Nevertheless, costs involved in breeding at a distant, northern location may exist, e.g. individuals might lack important adaptations to environmental factors other than timing, or lack sufficient local knowledge [17-19]. 

This adaptation-by-dispersal strategy seems especially suitable for highly mobile species like migratory birds which often suffer from mistiming due to climate change [2,20,21]. Here, we investigated fitness consequences of long-distance dispersal in a long-distance migrant, the pied flycatcher. In this species, long-distance dispersal (mostly natal dispersal) over hundreds of kilometers is unusual, although it seems to occur more often than previously thought [22]. However, fitness consequences of such events have not been studied before. In the Netherlands, this species has failed to advance arrival and egg-laying sufficiently to keep up with the advances in the timing of its major food source, caterpillars [23], which are important for ensuring nestling growth [24]. Because timing of insect abundance is later at higher latitudes [25], individuals arriving too late in their original breeding area could restore phenological matching by continuing migration further north and breed there. Benefits of dispersal to the north are however only expected during warm springs, because migrating and breeding too early can result in severe fitness costs (e.g. 26).

 In order to study the fitness consequences of breeding at a new, distant, northern site, we translocated pied flycatchers from The Netherlands to Southern Sweden and let birds breed under natural conditions. Our aims were to study: 1. if translocated birds are able to breed earlier than local birds; 2. their reproductive success relative to local breeders 3. the responses in stress physiology to the new environment; and 4. interpret those findings in relation to environmental circumstances. We hypothesized that, in warm springs, as they frequently occur in recent years, translocated flycatchers should have an advantage because they can reproduce earlier in the season compared to local birds, and that this results in higher breeding success. The benefits of reproducing relatively early might however be reduced because birds lack adaptations to other environmental factors. In a cold spring, we predict no advantage or even a disadvantage of translocated birds, as they might be arriving and breeding too early relative to the local phenology.

## Methods

### Ethics statement

All experimental procedures at the Swedish site were approved by the Malmö/Lund Animal Care Committee (permit no. M3-10). The import of pied flycatchers into Sweden and use of the temporary aviaries were approved by the Swedish Agricultural Board (permit nos. 30-4349/10 and 31-211/10, respectively). Permit to keep and subsequently release birds from the aviaries were granted by the Swedish Environmental Protection Agency (permit nos. 414-1314-10 and 414-1879-10) and the County Administrative Board of Skåne (permit no. 521-3703-10). All experimental procedures at the Dutch site were approved by the Animal Experiment Committee (DEC, permit no. 5588D). Staatsbosbeheer Dwingelderveld kindly allowed us to work in their forests.

### Study species

Pied flycatchers are long-distance migratory passerines breeding in temperate forests across Europe and wintering in sub-Saharan Western to Central Africa. They are a hole-nesting species that readily breeds in nest-boxes and defends only a small area around the nest. Pied flycatchers are single-brooded and parents usually provide bi-parental care to the young [27]. Their breeding habitat ranges from deciduous forests (often with oak *Quercus* spp. or birch *Betula* spp.) to mixed and coniferous forest (mostly consisting of pine *Pinus* spp.). 

### Study sites

The two study sites in which the experiment took place were located in Drenthe, The Netherlands (52° 50' N, 6° 22' E) and Vombs fure, east of Lund in Southern Sweden (55 ° 40' N, 13 ° 33' E), about 560 km apart. All nest boxes in The Netherlands had inner dimensions of 90*120*230 mm (width*depth*height) and an entrance hole 32 mm. Nest boxes in Sweden had inner dimensions of 101*145*228 mm and an entrance hole of 32 mm. The Swedish site has a delayed phenology compared to the Dutch site, with mean laying dates of pied flycatchers being on average (2007-2010) on 21^th^ May (± 0.54 SE, n=119) versus 5^th^ May (± 0.25 SE, n=887) in The Netherlands, a difference of 16 days. Mean clutch sizes hardly differed between sites in the years 2007-2010 (6.43 ± 0.11 SE, n= 883 for NL, and 6.45 ±0.08 SE, n=125 for Sweden).

The breeding habitat from which the Dutch birds were removed for translocation consisted mainly of oak trees (*Quercus robur* and the exotic *Q. rubra*), but often mixed with pine (*Pinus sylvestris*) and birch (*Betula* *spp*), while in Sweden, the main tree species were pine and birch with few oak trees. The control site in the Netherlands at which the birds were released was a mixed forest of oak and pine.

### Translocation

In spring 2010, we captured pied flycatchers just after pair-formation at the start of the nest-building phase at their nest box [28], in our Dutch study site (‘translocated group’, n= 48 individuals, and ‘Dutch control’ group, n= 30 individuals) as well as in Sweden (‘Swedish control’, n= 36 individuals). To control for the translocation, we treated local pairs both in the Dutch area of origin (“Dutch controls”) and from the Swedish release area (“Swedish controls”) in the same way. Birds that were moved from the Netherlands to Sweden (‘translocated group’) were transported by car during the night. During the transport, birds were housed individually in small cages (ca. 20x15x18cm) where food and water were provided and which were covered with a cheese cloth. The translocated group consisted of an early (n=11 pairs) and a late group (n=12 pairs plus two extra females) which were caught and translocated on two dates (25 April and 5 May). Those two dates are centered around the average date when most birds start nest-building as we needed sufficient numbers of nests of the same stage where we could capture birds. Individuals of the two control groups in The Netherlands and Sweden were captured during several days throughout the nest-building period and kept individually in transport cages over night at the nearby field stations (in Sweden, five birds were not kept overnight but released a few hours after capture). 

Individuals of all treatment groups were released in pairs into aviaries (ca 2x2x2 m, with a nest box) in the forest the next morning following capture [28]. Food (mealworms and small crickets) and water were provided *ad libitum* in the aviaries. The translocated group was released into outdoor aviaries in Sweden, which were located at a randomly chosen location within the forest study site (choice made among suitable habitat patches within the area). Control birds were moved into aviaries that were located several hundred meters away from the place of capture (from around 500 m up to 3 km in Sweden and up to 13 km in the Netherlands). In Swedish controls, 15 out of 18 pairs were original pairs. The remaining pairs were formed from birds being caught at different nest boxes. Of the Dutch controls, 12 out of 15 pairs were original pairs and of the translocated group 19 out of 23 were original pairs. After a period of 2 to 3 days, aviaries were opened to release birds without touching them and without removing the nest box. Food was continued being provided at the release site for more than a day to keep the birds attached to the local site. A previous pilot experiment, using a similar procedure, showed that all nine translocated females bred at the site of release more than 10 km from their original breeding site [28]. Some losses due to predation occurred during the aviary-phase (four birds of the translocated group, four of the Swedish control and five of the Dutch control group). 

Because birds moved more often than expected, after releasing them from the aviaries, nests were also included in the treatment group if only the respective female was manipulated in the described way, regardless of the male which was often an un-manipulated bird if the female moved to a different nest box (Swedish controls: 8 out of 14 males were un-manipulated; translocated: 7 out of 10 males; Dutch controls: 1 out of 8 males). In this paper, we therefore present data only on translocated females. Using only original pairs would have reduced the sample size substantially and females pairing up with local males also reflect more of the natural situation when females would disperse themselves.

### Measurements

#### 1. Timing & morphology

We recorded date of the first egg, clutch size and tarsus length and weight for nestlings and parents at a nestling age of 12 days (day of hatching = 0). We also calculated the interval (in days) between release from the aviary to laying of the first egg in females. 

#### 2. Stress physiology

At day 12 of nestling age, blood smears of female parents were made for analysis of white blood cells (heterophil to lymphocyte ratio, H/L) as an indicator of stress [29]. On each slide, 100 white blood cells were counted and assigned to the five different cell types: heterophils, lymphocytes, monocytes, eosinophils and basophils. We compared H/L ratios and proportions of monocytes in the blood of females, as monocytes should also increase in response to (immunological) stress [29]. Both parameters were highly significantly correlated (Pearson correlation coefficient, r = 0.57, df = 38, 95% confidence interval: 0.32 to 0.75, p<0.001), justifying the presentation here of only H/L ratios as a reliable measure of stress in the following results.

### Food abundance and nestling diet

We quantified seasonal changes in caterpillar abundance by using frass nets under trees (under three oak trees in NL and under two birch trees in S) to collect caterpillar droppings during the breeding season [5]. Traps were usually emptied every 3-4 days, but longer intervals occurred in some cases (up to 10 days, due to e.g. rainfall).

Data on nestling diet was collected with the use of nest box photos [24] to investigate differences between locations and treatments. Age of nestlings was between 8 and 13 days and nests were sampled only once for at least 90 minutes. Number of recorded prey items per nest varied between 32 and 166. From the photos we excluded all items which could not be identified (13% of all prey items), and calculated proportions of prey types from the remainder.

### Temperature

Temperature data were obtained from the freely available datasets of the European Climate Assessment & Dataset [30] from the closest available weather station to each study site (for NL: Station Eelde, 50 km from study site, for Sweden: Station Falsterbo, 50 km from study site). Mean spring temperature was calculated as the period between 50 days before until 30 days after the median hatch dates (calculated using hatch dates of the years 2007 and 2008 for Sweden and 2007-2010 for the Netherlands) for the two populations from 1980 to 2010 (Sweden: 19th April – 8th July; Netherlands: 2nd April – 21^st^ June). This was done to identify if the year of our experiment was as a relatively warm or cold year. 

### Statistical analysis

Statistical analysis was done using general linear models (function *glm*, package *stats*) and linear mixed models (function *lmer*, package *lme4*) in R 2.11.1 [31].. Final models were selected by backwards elimination of terms and using likelihood ratio tests. Multiple comparisons of treatment means (Tukey contrasts) were performed post-hoc using the function *glht* (package *multcomp*). H/L ratios were log-transformed to achieve a normal distribution of residuals. The model on nestling weights also contained tarsus length as covariate to control for the structural size of the bird and the model on number of fledglings contained laying date as covariate. Covariates (e.g. tarsus, date) were centered around their means.

## Results

### Temperatures, food abundance and nestling diet

The spring of 2010, the year of the experiment, was unusually cold: mean spring temperature was 0.95 °C lower (12.26 °C) than the 30-year trend-line in Sweden, and 1.89 °C lower (10.5 °C) than the trend in the Netherlands. This likely affected the outcome of our experiment as we expected benefits of long-distance dispersal mostly in relatively warm springs.

**Figure 1:**
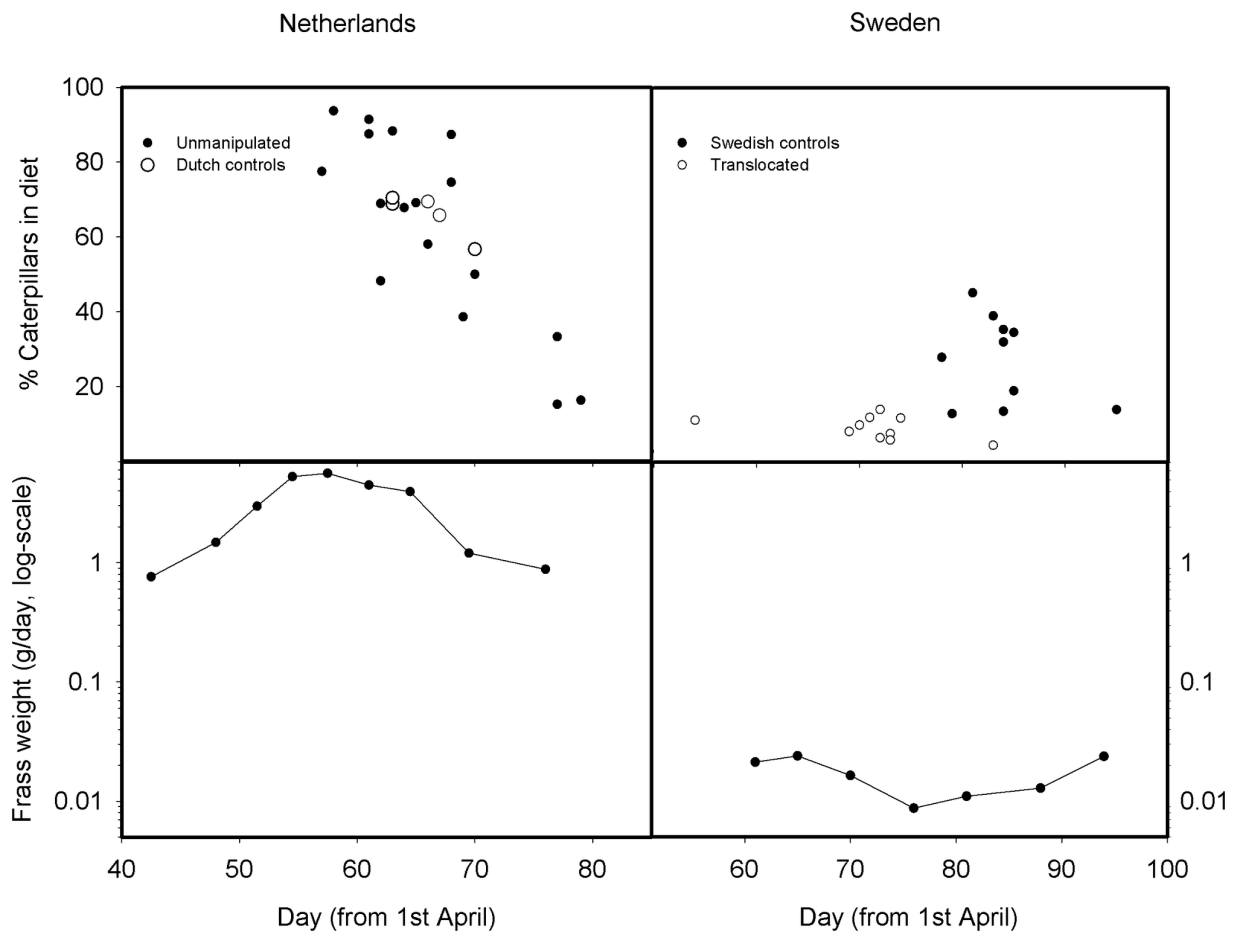
Percentage of caterpillars in nestling diet and caterpillar frass weights. Both nestling diet and frass weights (g per day per frass-net) are shown in relationship with date (for frass-nets: mean date between two sampling events), for Sweden and The Netherlands. Dutch broods showed a steep decline in the proportion of caterpillars in the diet with date, which was not the case for Swedish birds where proportions were generally lower.

Patterns of food abundance differed between locations: we found that the Dutch site showed a clear caterpillar peak (around 28 May), while caterpillar abundance in Sweden appeared rather constant over the breeding season (Figure 1). This difference is most likely related to the differences in habitat type between the two locations: for oak-mixed forests in The Netherlands, a caterpillar peak in spring is the typical pattern. In 2010, in The Netherlands, this peak occurred about 20 days later than in the three previous years (2007-2009) and was considerably lower than in the years before. Comparable data on caterpillar peaks during earlier years was missing for the Swedish study site. There was, however, a seasonal decline in nestling weights during previous years (data from 2007 and 2008, two relatively warm years, showing a decline of -0.048 and -0.068 g/day, respectively), and thus we assumed that, also in Sweden, late-breeding birds suffered from food-limitation. Although the total amount of caterpillars on trees between locations could not be quantified well with our method, the amounts of frass collected in the nets were generally much lower in Sweden compared to the Netherlands (Figure 1). 

The composition of nestling diets also suggested that less caterpillars were available in Sweden, with averages of 27.5 % for Swedish controls (n = 10), 9.3 % for translocated (n = 10), but 65.9 % for the Dutch nests (n = 17, mostly un-manipulated nests were used due to low sample size, Figure 1). Among the three most important prey types were, for Swedish controls, spiders (19.7 %) and beetles (12.6 %), for translocated nests beetles (26.3%) and spiders (16.5 %), and for Dutch controls beetles (11.7 %) and spiders (6.4 %). 

### Settlement success

 Settlement success of pied flycatchers differed among treatment groups: Of the translocated group, 10 out of the 23 released females (43%) were later recaptured as breeders in the release area in Sweden but only 5 out of 21 males (24%). Of the Swedish controls, 28 out of 32 released birds were recaptured later on as breeders (88%) and 19 out of 25 birds of the Dutch controls (76%). Both the translocated (6 out of 10 females) and the Swedish control birds (20 out of 28 individuals) often moved to another nest box within the study area (distances moved were between ca. 100 m and 2 km). This moving resulted in all cases in switching to a different partner (of the translocated group we only retained three original pairs and of the Swedish controls we retained two original pairs). The Dutch controls were more site-faithful after translocation, with 7 females and 8 males out of 19 birds staying with their designated nest box. There were no significant differences in body weight at capture, tarsus length, or age (t-tests, all p ≥ 0.5 ) between individuals that settled and those that did not. Of the settled females, three birds were 2^nd^ calendar year, three were older than that, and for the remaining four birds, age was unknown. The cold conditions in early spring could have affected settlement success of translocated birds in Sweden, e.g. by increasing mortality, but we found no difference in settlement success between the early (25^th^ April) and the late translocation-group (5^th^ May): 7 birds from the first catching cohort (out of 22) and 8 birds from the second cohort (out of 26) settled. 

### Timing, fitness parameters and physiology

**Figure 2:**
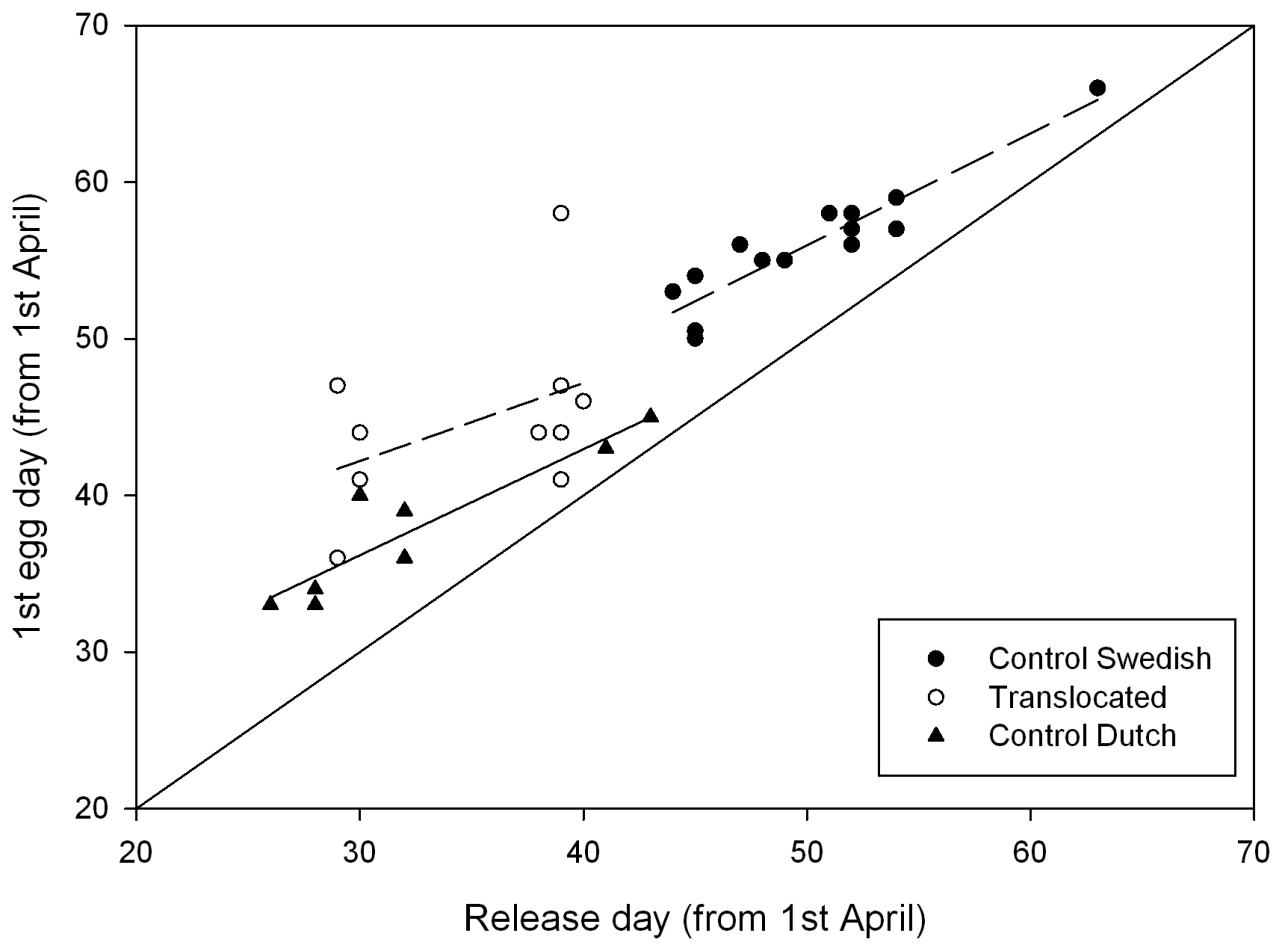
Release-day in relation to 1^st^ egg date. The relationship between the release day of female pied flycatchers from the aviaries and date of 1^st^ egg is shown for the three treatment groups. The duration between release from the aviaries and laying of the first egg varied from 2 to 19 days (all data points are above the x=y line) and declined with date, for each group. Each symbol indicates a nest, with separate regression lines for each treatment group. For graphical reasons, one data-point of Swedish controls was shifted +0.5 on the y-axis.

Translocated Dutch females started egg-laying 12 days (median date: 13^th^ May, n= 10 females) earlier than the local Swedish control females (mean date: 25^th^ May, n= 14 , Table 1) despite the relatively harsh weather conditions. Compared to Dutch controls (median date: 8^th^ May, n = 8 females), translocated females laid somewhat later (5.5 days, p = 0.04) which might be an effect of the lower temperatures in the North. Translocated females took longer between release and start of egg-laying than Dutch controls, and longer than Swedish controls (Figure 2), although the latter relation disappeared when controlling for date (Table 1). 

**Table 1:** Effect of treatment on the interval between release and first egg in females.

**Fixed effects**	**Estimates**	**Std. Errors**	**t-value**	**p-value**
Treatment: Control Dutch ^a^	2.38	1.51		^ab, ac^
Treatment: Control Swedish ^b^	9.10	1.35		^ba^
Treatment: Translocated ^c^	7.55	1.23		^ca^
Release day	-0.35	0.11	-3.10	0.004
**Excluded terms**				
Treatment*Release day				

Mean intervals were similar for translocated (10.7 days) and Swedish control groups (8.0 days) while they were significantly shorter for the Dutch controls (5.1 days). For treatments, significance (p<0.05) between groups, as calculated with a post-hoc test (Tuckey contrasts), is indicated by the combination of the superscripts a, b, c.

**Figure 3:**
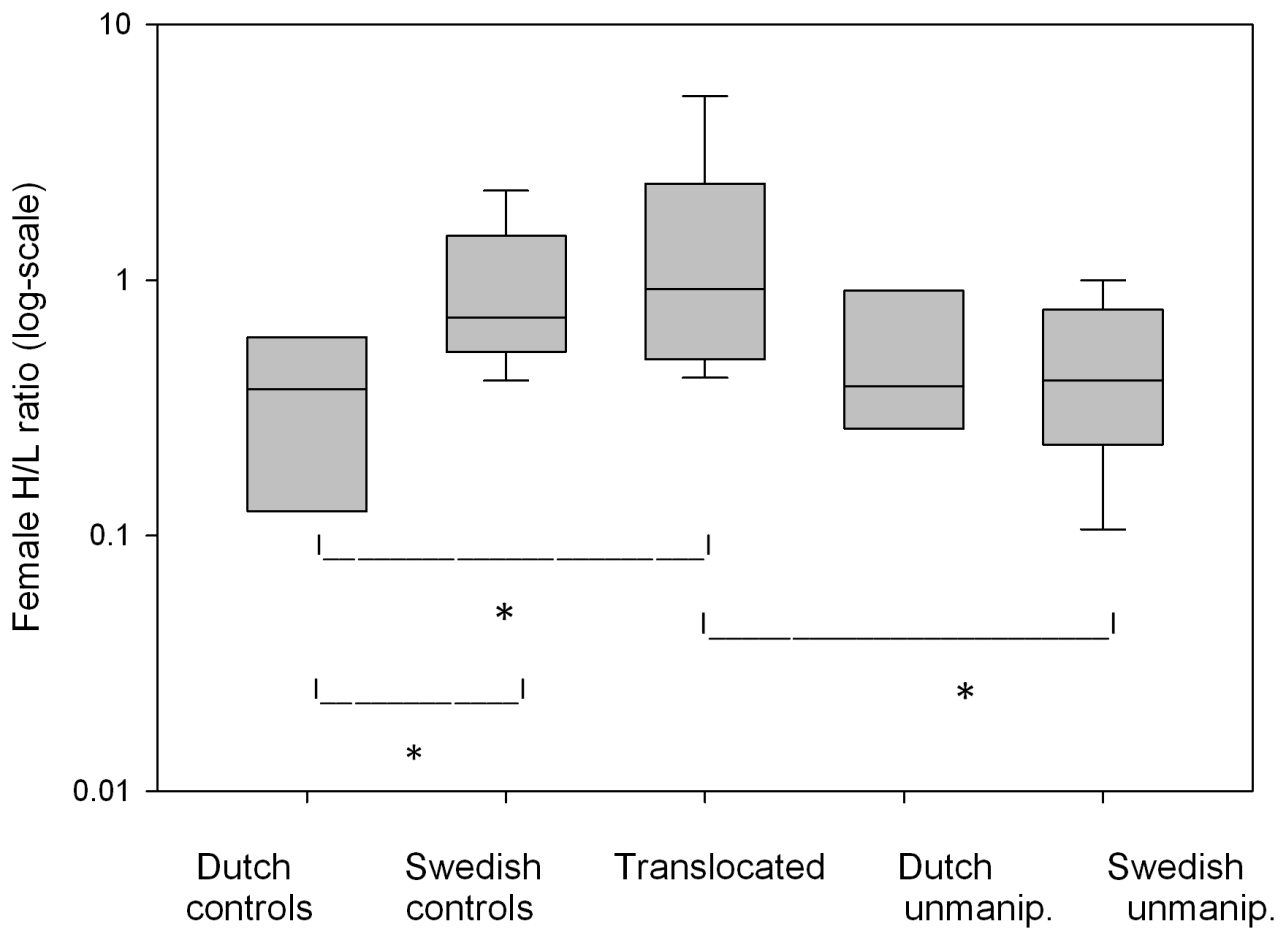
H/L ratios of female pied flycatchers. Values are shown for the three treatment groups and un-manipulated birds in the Netherlands and Sweden. Medians are shown, with boxes for the 25% to 75% percentiles and whiskers for 10% to 90% of the data. Higher values indicate more stress. H/L values are presented on a log-scale. Translocated and Swedish control females had significantly higher H/L values (means: 1.55 and 1.0, respectively) than the Dutch controls (mean: 0.40), while there was no significant difference between the two experimental groups breeding in Sweden. Significant differences (p<0.05) of post-hoc pairwise comparisons of groups are indicated by stars.

A general linear model with log-transformed H/L ratios (n = 40 females) for the treatment groups (including un-manipulated nests both in The Netherlands and Sweden) indicated a treatment effect while date had no significant effect on H/L ratios and was excluded in the final model. Subsequent post-hoc analysis of H/L ratios for treatment groups showed no difference between translocated Dutch females (median= 0.92) and control Swedish females (median = 1.03, estimate: 0.24 ± 0.37 SE, p = 0.97, Figure 3), but we found an effect of breeding location when comparing control groups: in Sweden, H/L ratios were higher, indicating more stress, compared to The Netherlands (median = 0.37, estimate: 1.23 ± 0.43 SE, p = 0.03). Furthermore, H/L ratios were lower for both Dutch and Swedish un-manipulated females compared to translocated females (significant effect only with Swedish un-manipulated females, estimate: -1.04 ± 0.38 SE, p = 0.047, Figure 3). 

Clutch sizes in Sweden differed between females of Dutch and Swedish origin. Although Swedish control females laid later in the season and clutch size usually declines with date [8], these females produced significantly larger clutches compared to translocated Dutch females (6.64 eggs ± 0.13 SE, n = 14 versus 5.90 eggs ± 0.23 SE, n =10, respectively; linear model estimate: 0.74 ± 0.27 SE, p = 0.015). 

We used two measures of reproductive success, the number of fledglings and fledgling condition (weight controlled for tarsus length). For fledgling production, we used two analyses: the first analysis used the number of fledglings as dependent, and treatment and laying date as fixed effects. For population growth it is irrelevant whether a high fledgling production results from larger clutches or higher per-egg fledging success. The final model contained only treatment as fixed effect (the effect of laying date was not significant, estimate: 0.027 ± 0.041 SE, p = 0.51; from linear model; normal error distribution seemed better than using poisson errors). Post-hoc analysis showed somewhat less fledglings from translocated nests (mean: 5.71 ± 0.26 SE) compared to Swedish control nests (mean: 6.29 ± 0.16 SE), but the difference was not significant (estimate: -0.59 ± 0.43 SE, p = 0.37), and significantly more fledglings in Swedish control nests compared to Dutch controls (mean for Dutch controls: 5.13 ± 0.61 SE; estimate: 1.16 ± 0.46 SE, p = 0.03, Tuckey contrasts). In the second analysis on fledgling production, we used the number of fledglings per number of eggs in a clutch as dependent, and including treatment and laying date as fixed effects, in order to test if there is also an effect of experimental treatment on the per-egg fledging success. Here, we found no significant difference among the treatment groups nor an effect of date (final model was the null model, y~1, using *glm* with binomial error distribution). 

For fledgling condition, we applied a linear mixed model with tarsus as a fixed effect and nest identity as random effect. We found no significant difference between Dutch control and translocated nests for fledgling condition (Table 2), but translocated nests had lower body weights than Swedish control nests (Table 2). 

**Table 2:** Effect of treatment on fledgling condition.

**Fixed effects**	**Estimates**	**Std. Errors**	**t-value**	**p-value**
Treatment: Control Dutch ^a^	13.803	0.222		^a^
Treatment: Control Swedish ^b^	14.188	0.163		^bc^
Treatment: Translocated ^c^	13.568	0.195		^cb^
Tarsus	0.938	0.134	7.018	< 0.001
**Random effect**	**Std. Deviation**			
Nest	0.563			
**Excluded terms**				
Date				
# Fledglings				
Treatment*Date				

Data on fledgling weights of 180 nestlings from 32 nests was used, for the three treatment groups, and controlled for tarsus length. For the treatment groups, a significant difference (p<0.05, from post-hoc test) between groups is indicated by the combination of the superscripts a, b, c.

In summary, we found no evidence that translocated females reproduced better than control Dutch birds (neither in number nor condition of the fledglings), which we initially expected given the observed mismatch in The Netherlands. Instead, translocated Dutch females produced a somewhat lower number of fledglings that were in lower condition, compared to Swedish females. However, female stress levels (H/L ratios) did not differ significantly between translocated and Swedish control groups, but values tended to be lower in Dutch control birds (Figure 3). Against our expectations about the importance of timing, there was no significant negative effect of date on the number of fledglings nor on fledgling weights (Table 2).

## Discussion

Climate change has resulted in a stronger advance of caterpillars than the hatching date of pied flycatchers [32], and our interest in this paper is whether long-distance dispersal to the north could be an option for the birds to improve their timing relative to the local caterpillar peak. With our experimental approach we showed that translocated females were indeed able to start egg-laying earlier compared to local birds at the northern location which could be beneficial in a warm spring. However, in this study, we could not find any advantage of breeding early, in terms of reproductive success, for broods of translocated individuals. We suggest that this lack of advantage mainly results from the exceptionally cold weather and the lack of an early caterpillar peak in the year of the experiment. 

Translocated females took longer between release and start of egg laying than Dutch controls, and longer than Swedish controls. A likely explanation for this is that flycatcher females, irrespective of breeding area, delay laying relative to arrival during adverse weather conditions due to difficulties in accumulating resources for egg production [33]. Therefore, the low temperatures most likely hampered the start of laying in translocated females in this cold year. 

The interval which females took between release and egg laying showed a similar decline with date for translocated and Swedish control females which suggests that the origin of birds has little influence on the response to the environment and that translocated females behaved naturally. We think that the earlier laying dates of translocated females resulted mainly from their earlier arrival in Sweden, compared to Swedish control birds, which enabled them to build a nest and accumulate resources for egg-laying, earlier than local females. Also, females from a Southern origin might be advanced in their internal physiology and thus able to lay earlier. But regardless of the mechanism, we would expect naturally dispersing individuals to show a similar advance in laying dates as our translocated females. Additional evidence for the natural behaviour of translocated females came from measurements of female stress physiology [29,34] during the late brood-rearing phase. H/L ratios indicated that our manipulation did not stress birds over a long period. The apparently higher stress that manipulated birds experienced in Sweden might be related to the lower settlement probability of females at the nest-box of release, compared to Dutch controls, as searching for a new nesting site could be stressful [19]. 

Overall, our data did not show the expected fitness benefits for translocated females. This could be interpreted as a general cost of long-distance dispersal through insufficient knowledge with the local area or a lack of locally adapted gene-complexes. We argue that most likely this is a particular year effect explained by the relatively low temperatures in combination with low food abundance. Translocated females might have been resource-limited early in this season and therefore produced smaller clutches [23], although this does not explain the smaller clutch sizes also in Dutch controls. If populations use different wintering areas, a carry-over effect of wintering habitat quality on clutch size could result in such differences between populations [35]. The better condition of fledglings from Swedish controls compared to fledglings from translocated females may indicate a resident advantage of Swedish birds potentially depending on local adaptations. Thus, such adaptations might be visible during a year with no obvious advantage of early breeding. However , even in a relatively cold spring, females are able to reproduce successfully after dispersal to the North, as evidenced by the fact that three male offspring of translocated females (out of 57 fledglings) recruited into the Swedish population in the following year and produced fledglings (A. Nord, personal observation).

The lack of a clear caterpillar peak at the Swedish site also suggests that early breeders did not profit from high caterpillar abundance. A crucial question is therefore, how important timing of breeding is for the studied populations, as the habitats in Sweden and The Netherlands differ strongly in seasonality of caterpillar abundance [24,36]. Birds breeding in the North might rely more on other, less seasonal prey types than on the tree-dwelling caterpillar species which we measured. Therefore, selection pressures for early breeding are likely to be higher in the Dutch pied flycatcher population, where birds breed predominately in oak forests with a narrow caterpillar peak. Thus, any dispersal to less seasonal habitats might therefore be advantageous for this species. However, environmental conditions, other than caterpillar abundance, may generally favour early breeding in less seasonal habitat and also at the northern study site [37], but this was not the case in the harsh weather during our experiment. 

With our experimental approach we aimed to rule out problems in studies of dispersal such as the non-randomness of dispersal [38], which can lead to an overestimation of dispersal costs if e.g. dispersing birds are of lower quality. Unlike the birds in a previous pilot study [28], settlement success of translocated birds was rather low, especially for males. We could however not find differences between birds that settled versus those that did not. We could not quantify the cost of migrating the additional distance to the North, but we assume that this distance could be covered under natural conditions in about two nights of migration [16], and that travel costs for a long-distance migrant such as the pied flycatcher are relatively low, especially if spring phenology is more advanced. In line with this, two of the 44 translocated birds returned to the Dutch study site within the same breeding season (C. Both, personal observation) indicating that birds can cover distances of several hundred kilometres rather easily within a few days. 

In this study system, we speculate that fitness benefits of latitudinal dispersal are likely to be high during warm springs. Our results suggest that pied flycatchers can successfully introduce their early breeding phenotype after dispersing to more northern areas. However, our findings remain preliminary as the experiment was only performed in a single, cold year. Moreover, we only studied one aspect of individual fitness, reproductive success, and could not quantify survival well (only anecdotal evidence). A complete picture also requires knowledge about heritabilities of the traits under study- not just within populations as is commonly studied, but also between populations-, to estimate the potential for adaptation through gene flow. 

Theoretically, strong, undirected gene flow might act against local adaptation because it swamps adaptive gene-complexes [39], but heterogeneous dispersal as mimicked in this study can indeed promote micro-evolution when new genetic variation is introduced needed for adaptation to novel ecological conditions [40]. Successful adaptation will generally depend on a balance between benefits of the new, introduced traits and the costs of breaking up other important adaptations. As long-distance dispersal occasionally occurs in long-distance migrants [22,10], our findings imply that research should be concentrating on the causes of phenotypic variation between populations [41], and how this could affect the ability and speed of a species’ adaptation to climate change.

## References

[1] Parmesan C, Yohe G (2003) A globally coherent fingerprint of climate change impacts across natural systems. Nature 421: 37-42. doi:10.1038/nature01286. PubMed: 12511946. 12511946

[2] Both C, Bouwhuis S, Lessells CM, Visser ME (2006) Climate change and population declines in a long-distance migratory bird. Nature 441: 81-83. doi:10.1038/nature04539. PubMed: 16672969. 16672969

[3] Thackeray SJ, Sparks TH, Frederiksen M, Burthe S, Bacon PJ et al. (2010) Trophic level asynchrony in rates of phenological change for marine, freshwater and terrestrial environments. Global Change Biol 16: 3304-3316. doi:10.1111/j.1365-2486.2010.02165.x.

[4] Martin TE (1987) Food as a limit on breeding birds: A life-history perspective. Annual Rev Ecol Syst 18: 453-487. doi:10.1146/annurev.es.18.110187.002321.

[5] Visser ME, Holleman LJM, Gienapp P (2006) Shifts in caterpillar biomass phenology due to climate change and its impact on the breeding biology of an insectivorous bird. Oecologia 147: 164–72. PubMed: 16328547. 16328547 10.1007/s00442-005-0299-6

[6] Blows MW, Hoffmann AA (2005) A reassessment of genetic limits to evolutionary change. Ecology 86: 1371-1384. doi:10.1890/04-1209.

[7] Edelaar P, Siepielski AM, Clobert J (2008) Matching habitat choice causes directed gene flow: A neglected dimension in evolution and ecology. Evolution 62: 2462-2472. doi:10.1111/j.1558-5646.2008.00459.x. PubMed: 18637835. 18637835

[8] Thomas DW, Blondel J, Perret P, Lambrechts MM, Speakman JR (2001) Energetic and fitness costs of mismatching resource supply and demand in seasonally breeding birds. Science 291: 2598-2600. doi:10.1126/science.1057487. PubMed: 11283370. 11283370

[9] Bowler DE, Benton TG (2005) Causes and consequences of animal dispersal strategies: relating individual behaviour to spatial dynamics. Biol Rev Camb Philos Soc 80: 205-225. doi:10.1017/S1464793104006645. PubMed: 15921049. 15921049

[10] Winkler DW, Wrege PH, Allen PE, Kast TL, Senesac P et al. (2005) The natal dispersal of tree swallows in a continuous mainland environment. J Anim Ecol 74: 1080-1090. doi:10.1111/j.1365-2656.2005.01007.x.

[11] Garant D, Kruuk LEB, Wilkin TA, McCleery RH, Sheldon BC (2005) Evolution driven by differential dispersal within a wild bird population. Nature 433: 60-65. doi:10.1038/nature03051. PubMed: 15635409. 15635409

[12] Liedvogel M, Cornwallis CK, Sheldon BC (2012) Integrating candidate gene and quantitative genetic approaches to understand variation in timing of breeding in wild tit populations. J Evol Biol 25: 813-823. doi:10.1111/j.1420-9101.2012.02480.x. PubMed: 22409177. 22409177

[13] Silverin B, Massa R, Stokkan KA (1993) Photoperiodic adaptation to breeding at different latitudes in great tits. Gen Comp Endocrinol 90: 14-22. doi:10.1006/gcen.1993.1055. PubMed: 8504918. 8504918

[14] Perfito N, Jeong SY, Silverin B, Calisi RM, Bentley GE et al. (2012) Anticipating spring: Wild populations of great tits (*Parus* *major*) differ in expression of key genes for photoperiodic time measurement. PLOS ONE 7: e34997. doi:10.1371/journal.pone.0034997. PubMed: 22539953. 22539953 PMC3334499

[15] Lehtonen PK, Laaksonen T, Artemyev AV, Belskii E, Both C et al. (2009) Geographic patterns of genetic differentiation and plumage colour variation are different in the pied flycatcher (*Ficedula* *hypoleuca*). Mol Ecol 18: 4463-4476. doi:10.1111/j.1365-294X.2009.04364.x. PubMed: 19796331. 19796331

[16] Both C (2010) Flexibility of timing of avian migration to climate change masked by environmental constraints en route. Curr Biol 20: 243-248. doi:10.1016/j.cub.2009.11.074. PubMed: 20116248. 20116248

[17] Brown CR, Brown MB, Brazeal KR (2008) Familiarity with breeding habitat improves daily survival in colonial cliff swallows. Anim Behav 76: 1201-1210. doi:10.1016/j.anbehav.2008.03.028. PubMed: 19802326. 19802326 PMC2598429

[18] Clobert J, Nichols JD, Danchin E, Dhont A (2001) Dispersal. Oxford University Press, Oxford.

[19] Part T (1995) The importance of local familiarity and search costs for age-biased and sex-biased philopatry in the collared flycatcher. Anim Behav 49: 1029-1038. doi:10.1006/anbe.1995.0132.

[20] Møller AP, Rubolini D, Lehikoinen E (2008) Populations of migratory bird species that did not show a phenological response to climate change are declining. Proc Natl Acad Sci U S A 105: 16195-16200. doi:10.1073/pnas.0803825105. PubMed: 18849475. 18849475 PMC2571031

[21] Visser ME (2008) Keeping up with a warming world; assessing the rate of adaptation to climate change. Proc Biol Sci 275: 649-659. doi:10.1098/rspb.2007.0997. PubMed: 18211875. 18211875 PMC2409451

[22] Both C, Robinson RA, van der Jeugd HP (2012) Long-distance dispersal in migratory pied flycatchers *Ficedula* *hypoleuca* is relatively common between the UK and the Netherlands. J Avian Biol 43: 193-197. doi:10.1111/j.1600-048X.2012.05721.x.

[23] Both C, Visser ME (2005) The effect of climate change on the correlation between avian life-history traits. Global Change Biol 11: 1606-1613. doi:10.1111/j.1365-2486.2005.01038.x.

[24] Burger C, Belskii E, Eeva T, Laaksonen T, Mägi M et al. (2012) Climate change, breeding date and nestling diet: How temperature differentially affects seasonal changes in pied flycatcher diet depending on habitat variation. J Anim Ecol 81: 926-936. doi:10.1111/j.1365-2656.2012.01968.x. PubMed: 22356622. 22356622

[25] Smith KW, Smith L, Charman EBK, Burgess M, Dennis C et al. (2011) Large-scale variation in the temporal patterns of the frass fall of defoliating caterpillars in oak woodlands in Britain: Implications for nesting woodland birds. Bird Study 58: 506-511. doi:10.1080/00063657.2011.616186.

[26] Brown CR, Brown MB (2000) Weather-mediated natural selection on arrival time in cliff swallows (*Petrochelidon* *pyrrhonota*). Behav Ecol Sociobiol 47: 339-345. doi:10.1007/s002650050674.

[27] Lundberg A, Alatalo RV (1992) The pied flycatcher. Poyser, London.

[28] Burger C, Both C (2011) Translocation as a novel approach to study effects of a new breeding habitat on reproductive output in wild birds. PLOS ONE 6: e18143. doi:10.1371/journal.pone.0018143. PubMed: 21479183. 21479183 PMC3068174

[29] Davis AK, Maney DL, Maerz JC (2008) The use of leukocyte profiles to measure stress in vertebrates: A review for ecologists. Funct Ecol 22: 760-772. doi:10.1111/j.1365-2435.2008.01467.x.

[30] Klein Tank AMG, Wijngaard JB, Können GP, Böhm R, Demarée G et al. (2002) Daily dataset of 20th-century surface air temperature and precipitation series for the european climate assessment. Int J Climatol 22: 1441-1453. doi:10.1002/joc.773.

[31] RDevelopmentCoreTeam (2008) R: A language and environment for statistical computing. R Foundation for Statistical Computing, Vienna, Austria.

[32] Both C, Van Asch M, Bijlsma RG, van den Burg AB, Visser ME (2009) Climate change and unequal phenological changes across four trophic levels: Constraints or adaptations? J Anim Ecol 78: 73–83. doi:10.1111/j.1365-2656.2008.01458.x. PubMed: 18771506. 18771506

[33] Eeva T, Veistola S, Lehikoinen E (2000) Timing of breeding in subarctic passerines in relation to food availability. Can J Zool 78: 67-78. doi:10.1139/z99-182.

[34] Wikelski M, Cooke SJ (2006) Conservation physiology. Trends Ecol Evol 21: 38-46. doi:10.1016/j.tree.2005.10.018. PubMed: 16701468. 16701468

[35] Norris D, Marra P, Kyser T, Sherry T, Ratcliffe L (2004) Tropical winter habitat limits reproductive success on the temperate breeding grounds in a migratory bird. Proc R Soc of London B-Biol Sci 271: 59-64. doi:10.1098/rspb.2003.2569. PMC1691559 15002772

[36] Both C, Van Turnhout CAM, Bijlsma RG, Siepel H, Van Strien AJ et al. (2010) Avian population consequences of climate change are most severe for long-distance migrants in seasonal habitats. Proc R Soc of London B-Biol Sci 277: 1259-1266. doi:10.1098/rspb.2009.1525. PubMed: 20018784. PMC2842804 20018784

[37] Dunn PO, Winkler DW, Whittingham LA, Hannon SJ, Robertson RJ (2011) A test of the mismatch hypothesis: How is timing of reproduction related to food abundance in an aerial insectivore? Ecology 92: 450-461. doi:10.1890/10-0478.1. PubMed: 21618924. 21618924

[38] Doligez B, Pärt T (2008) Estimating fitness consequences of dispersal: A road to 'know-where'? non-random dispersal and the underestimation of dispersers' fitness. J Anim Ecol 77: 1199-1211. doi:10.1111/j.1365-2656.2008.01446.x. PubMed: 18808435. 18808435

[39] Lenormand T (2002) Gene flow and the limits to natural selection. Trends Ecol Evol 17: 183-189. doi:10.1016/S0169-5347(02)02497-7.

[40] Paradis E, Baillie SR, Sutherland WJ, Gregory RD (1998) Patterns of natal and breeding dispersal in birds. J Anim Ecol 67: 518-536. doi:10.1046/j.1365-2656.1998.00215.x.

[41] Coltman DW (2005) Evolutionary genetics - differentiation by dispersal. Nature 433: 23-24. doi:10.1038/433023a. PubMed: 15635394. 15635394

